# Percutaneous Transhepatic Variceal Embolization as a Therapeutic Bridge for Variceal Bleeding: An Initial Alternative in the Absence of Endoscopy or Transjugular Intrahepatic Portosystemic Shunt (TIPS)

**DOI:** 10.7759/cureus.102492

**Published:** 2026-01-28

**Authors:** Oscar F Vargas, Juliana Salcedo-Mesa, Laura Camila Ortiz Layton, William Alvarez

**Affiliations:** 1 Interventional Radiology, Hospital Departamental de Villavicencio, Villavicencio, COL

**Keywords:** case reports, embolization, esophageal varices, gastric varices, gastrointestinal hemorrhage, hematemesis, liver cirrhosis, portal hypertension

## Abstract

Variceal gastrointestinal bleeding in patients with liver cirrhosis is a potentially fatal complication that requires prompt hemostatic control. Standard management includes vasoactive therapy, antibiotic prophylaxis, and urgent endoscopic intervention, with transjugular intrahepatic portosystemic shunt reserved for selected high-risk or refractory cases. However, in resource-limited settings, timely access to endoscopy or definitive portal decompression may be delayed or unavailable, increasing the risk of hemodynamic deterioration and early mortality. We report the case of a 64-year-old woman with cirrhosis who developed hypovolemic shock secondary to gastroesophageal variceal hemorrhage and was treated with percutaneous transhepatic variceal embolization (PTVE) using a combined coil and ethylene-vinyl alcohol copolymer (Onyx) strategy, achieving immediate hemostasis and hemodynamic stabilization. This case highlights PTVE as a feasible bridge therapy in acute variceal bleeding when endoscopic or definitive portal decompression strategies are delayed or unavailable.

## Introduction

Esophageal varices occur when portal pressure exceeds 10 mmHg and may be present in 50% of patients with cirrhosis [1,2]. Variceal upper gastrointestinal bleeding is one of the most severe and life-threatening complications of portal hypertension, representing high morbidity and mortality worldwide [1]. Current international guidelines recommend early resuscitation followed by urgent endoscopic evaluation and therapy with vasoactive agents and antibiotic prophylaxis [2,3].

However, in many low- and middle-income countries with limited resources, immediate endoscopy is not always available. Delays in definitive hemorrhage control increase the risk of hemodynamic instability, recurrent bleeding, and early death [4].

For patients with refractory bleeding or those at high risk of treatment failure, transjugular intrahepatic portosystemic shunt (TIPS) is an established rescue strategy [4]. Nevertheless, TIPS requires specialized expertise and referral to high-complexity centers [4]. Therefore, urgent endoscopy may not be immediately available. For these reasons, there is a need for alternative strategies to stabilize critically ill patients while definitive treatments become feasible.

Percutaneous transhepatic variceal embolization (PTVE) is an alternative hemostatic approach. It may serve as a bridge to stabilize critically ill patients while definitive therapy becomes available or transfer to a high-complexity care center is arranged [5]. Although it is not the first-line treatment for variceal hemorrhage, there is increasing evidence of its utility when endoscopy or TIPS is delayed, contraindicated, or logistically infeasible [5]. Reducing transfusion requirements by selectively targeting arterial inflow to the bleeding variceal territory [5].

In this report, we describe the clinical course and endovascular management of a patient with hemodynamically significant esophageal variceal bleeding who underwent embolization as an initial therapy when endoscopy or TIPS were not immediately available. This case highlights the potential role of embolization as a practical, life-saving bridge therapy in resource-limited or time-sensitive settings.

## Case presentation

A 64-year-old woman with a history of cryptogenic liver cirrhosis and type 2 diabetes mellitus presented to the emergency department with a 1-day history of epigastric abdominal pain and five episodes of hematemesis. On admission, vital signs were: blood pressure 127/56 mmHg, heart rate 113 beats per minute, respiratory rate 18 breaths per minute, temperature 36°C, BMI 27.29, and Glasgow Coma Scale 15/15. Relevant past history included endoscopic band ligation of esophageal varices two years earlier and chronic treatment with sitagliptin-metformin, propranolol, and omeprazole.

Initial laboratory evaluation showed moderate anemia, mild thrombocytopenia, prolonged coagulation parameters, hypoalbuminemia, and preserved renal function (Table 1). A diagnosis of upper gastrointestinal bleeding in a cirrhotic patient was established. Initial management included intravenous fluids, proton pump inhibitor therapy, antiemetics, terlipressin, and continuous monitoring. Two units of packed red blood cells were reserved. Urgent endoscopy was not available at that time.

**Table 1:** Laboratories on admission and during the 72 hours of follow-up Serial laboratory investigations are summarized in this table. Laboratory parameters obtained on admission and during the first 72 hours of clinical follow-up are presented. The table highlights the evolution of anemia and thrombocytopenia during the melena episode and subsequent partial recovery after treatment. Dashes (—) indicate unavailable data.

Parameter	Reference range	On admission	During melena (72 h)	Post-treatment
Hemoglobin (g/dL)	12.0–16.0	9.5	6.8–7.3	7.7–8.4
Hematocrit (%)	36–46	29	23	27
Platelets (/µL)	150,000–450,000	148,000	81,000	135,000
White blood cell count (/µL)	4,000–11,000	9,170	4,890	7,010
Prothrombin time (s)	11–13.5	14.8	15.1	14.3
Activated partial thromboplastin time (s)	25–35	22.5	22.5	21.8
International normalized ratio (s)	0.8–1.2	1.55	1.26	1.19
Creatinine (mg/dL)	0.5–1.1	0.33	0.26	0.4
Blood urea nitrogen (mg/dL)	7–20	27.4	8.7	7.3
Total bilirubin (mg/dL)	0.2–1.2	0.74	—	—
Sodium (mmol/L)	135–145	137	—	—
Albumin (g/dL)	3.5–5.0	2.85	—	—

On the first day of hospitalization, the patient developed recurrent massive upper gastrointestinal bleeding with hemodynamic deterioration consistent with hypovolemic shock, prompting urgent activation of the Interventional Radiology team. An endovascular procedure was planned as first-line hemostatic therapy given the lack of immediate endoscopic access and the cirrhotic context. Transhepatic portography was performed, demonstrating multiple active variceal channels with evidence of bleeding. Superselective catheterization and embolization of three variceal pedicles were performed using coils, followed by Onyx injection, achieving complete occlusion without intraoperative complications.

According to the protocol, the patient was admitted to the intensive care unit. Mechanical ventilation was not required; however, vasopressor support and intravenous insulin were administered due to moderate diabetic ketoacidosis in the context of hemorrhagic shock. During the next 72 hours, she had one episode of melena associated with a further decrease in hemoglobin and progressive thrombocytopenia (Table 1). This was managed with transfusion of one unit of packed red blood cells and supportive care. As the patient improved, she tolerated oral intake, had no evidence of encephalopathy or respiratory compromise, and was started on propranolol and spironolactone after ultrasonographic evidence of ascites was noted. A therapeutic paracentesis was performed, draining 2,000 mL of clear fluid. Hemoglobin and platelet counts partially recovered, while renal function and coagulation parameters remained stable (Table 1).

Six days after admission, follow-up endoscopy revealed grade I esophageal varices without red wale signs, hypertensive corporo-antral gastropathy, and a 5-mm Forrest III antral ulcer, with no active bleeding. The patient remained hemodynamically stable, afebrile, and without recurrent gastrointestinal bleeding. Given her Child-Pugh B cirrhosis, portal hypertension, and recent variceal bleeding episode successfully controlled by embolization, the multidisciplinary team considered her a candidate for definitive portal decompression with TIPS. She was transferred in stable condition to a tertiary care center for TIPS and continued follow-up by gastroenterology and interventional radiology. Surveillance endoscopy four months later documented progression to grade II esophageal varices without stigmata of recent bleeding and chronic antral erythematous gastritis, supporting the need for ongoing nonselective beta-blocker therapy and periodic endoscopic surveillance.

Endovascular technique

**Figure 1:**
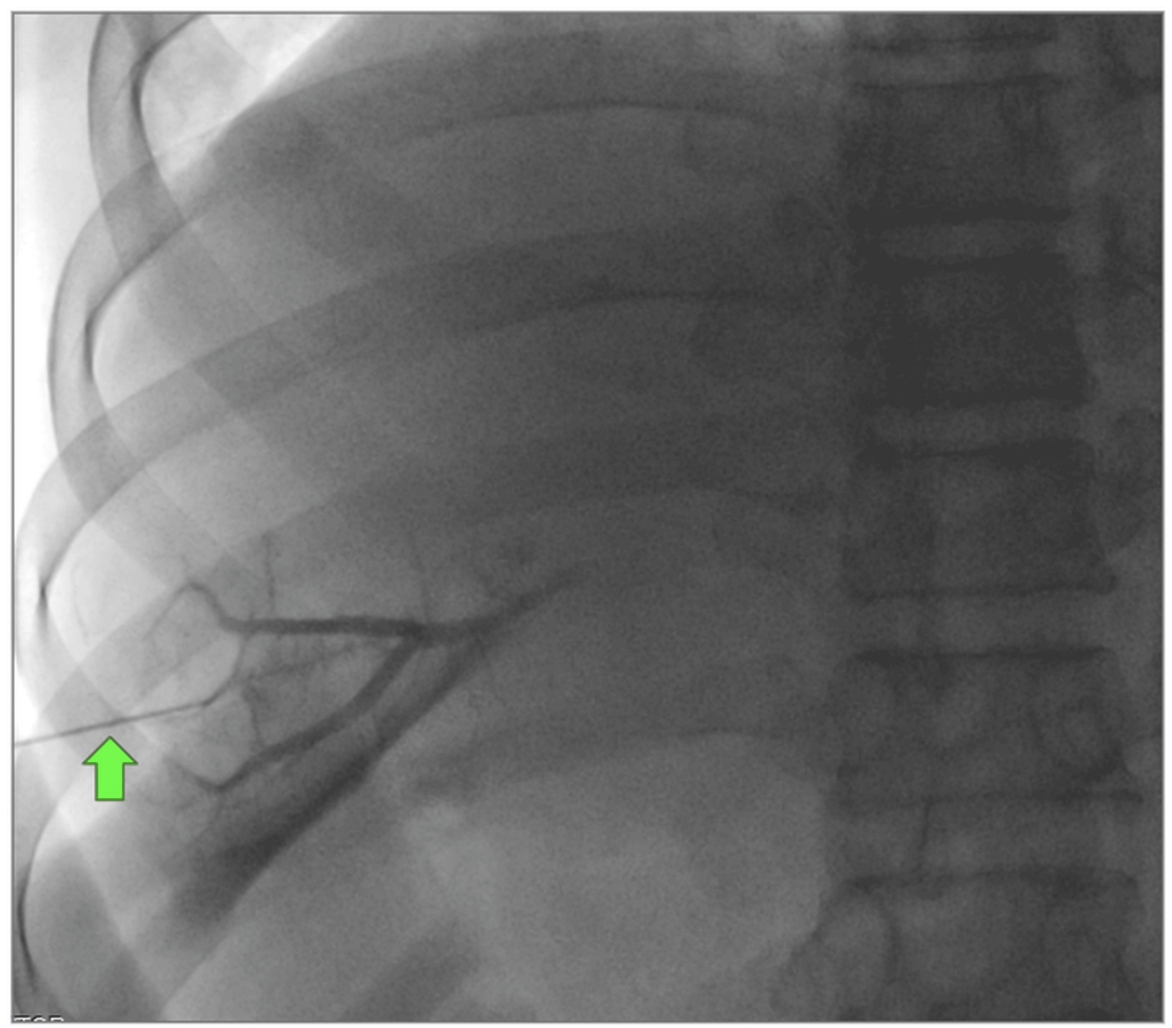
Portography via percutaneous transhepatic puncture of the portal venous system Percutaneous puncture of a peripheral portal vein was performed using the AccuStick™ II Introducer System. A Chiba needle was used to access the portal system, through which a micro guidewire was advanced. Contrast medium was injected to confirm portal venous anatomy. Green arrow: Chiba needle.

**Figure 2:**
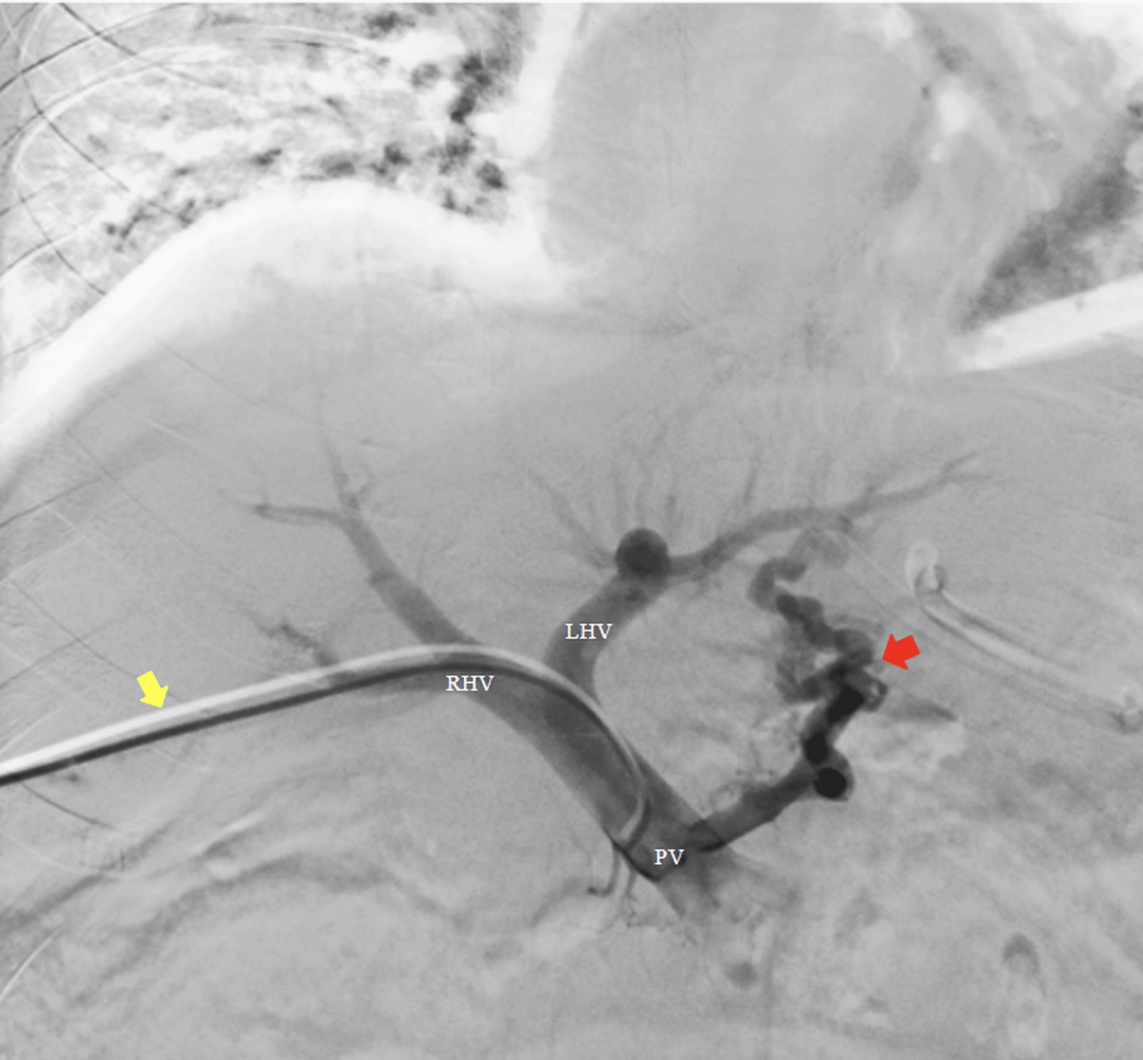
Portography via percutaneous transhepatic puncture of the portal venous system using a Cobra catheter Over the hydrophilic guidewire, a 5-Fr vascular introducer sheath was placed. Through the introducer sheath, a regular Cobra catheter was advanced. Portography was performed using a power injector with 5 mL of contrast at 200 psi (pounds per square inch). LHV: left hepatic vein. RHV: right hepatic vein. PV: portal vein. Yellow arrow: transhepatic Cobra catheter. Red arrow: gastroesophageal varices.

Under general anesthesia, percutaneous ultrasound-guided transhepatic portal vein access was obtained through a peripheral portal vein using an AccuStick™ introducer system (Boston Scientific, Marlborough, MA, USA) set, followed by placement of a 5-F introducer (Figure 1). A 5-F Cobra Glidecath catheter (Terumo, Tokyo, Japan) was advanced over a hydrophilic 0.035 x 260 cm guide wire. Transhepatic portography (10 mL/s, 10frames/seg, 200 psi) demonstrated multiple opacified gastroesophageal varices consistent with gastroesophageal varices type 1, with early venous filling consistent with active variceal inflow (Figure 2).

**Figure 3:**
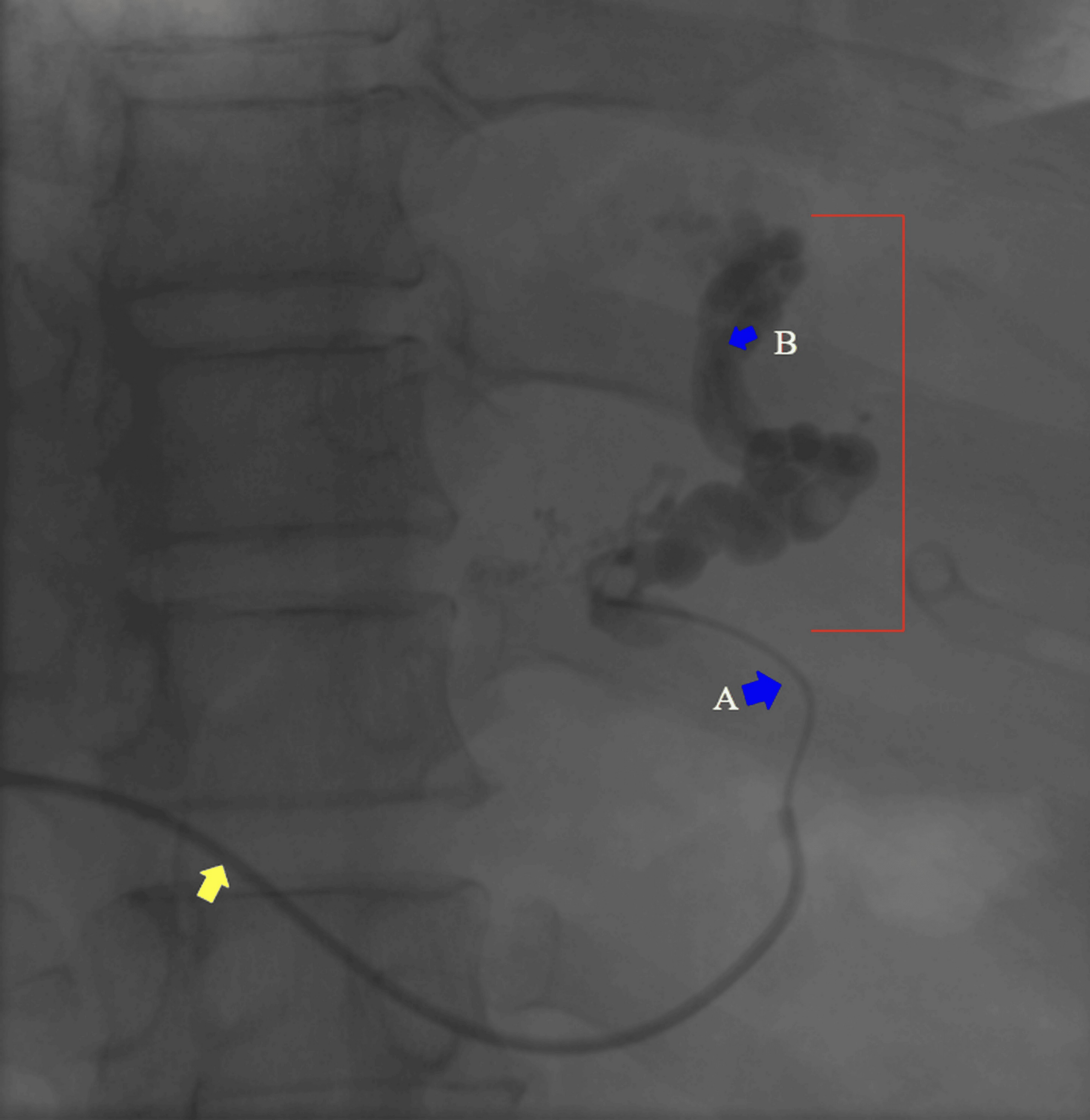
Selective injection into a gastroesophageal variceal feeder vein using a Progreat microcatheter Using a Progreat microcatheter, the gastroesophageal varices were selectively catheterized, advancing from the most distal achievable position to a proximal segment. Contrast medium was injected to opacify the gastroesophageal varices. Yellow arrow: transhepatic Cobra catheter. Blue arrows: (A) proximal position of the Progreat microcatheter; (B) most distal position achievable with the Progreat microcatheter. Red lines: gastroesophageal varices.

**Figure 4:**
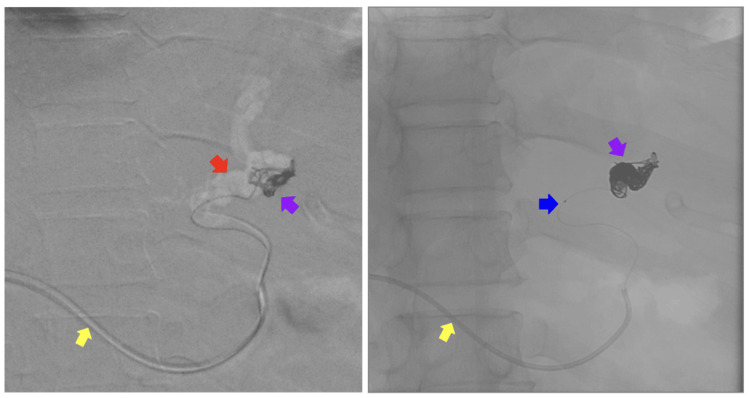
Gastrointestinal phlebography during coil deployment A large coil was deployed in the most distal segment of the varix, creating a coil cast over the Progreat microcatheter. Yellow arrow: transhepatic Cobra catheter. Blue arrow: radiopaque guide of the Progreat microcatheter. Purple arrows: coil deployment. Red arrow: gastroesophageal varices.

**Figure 5:**
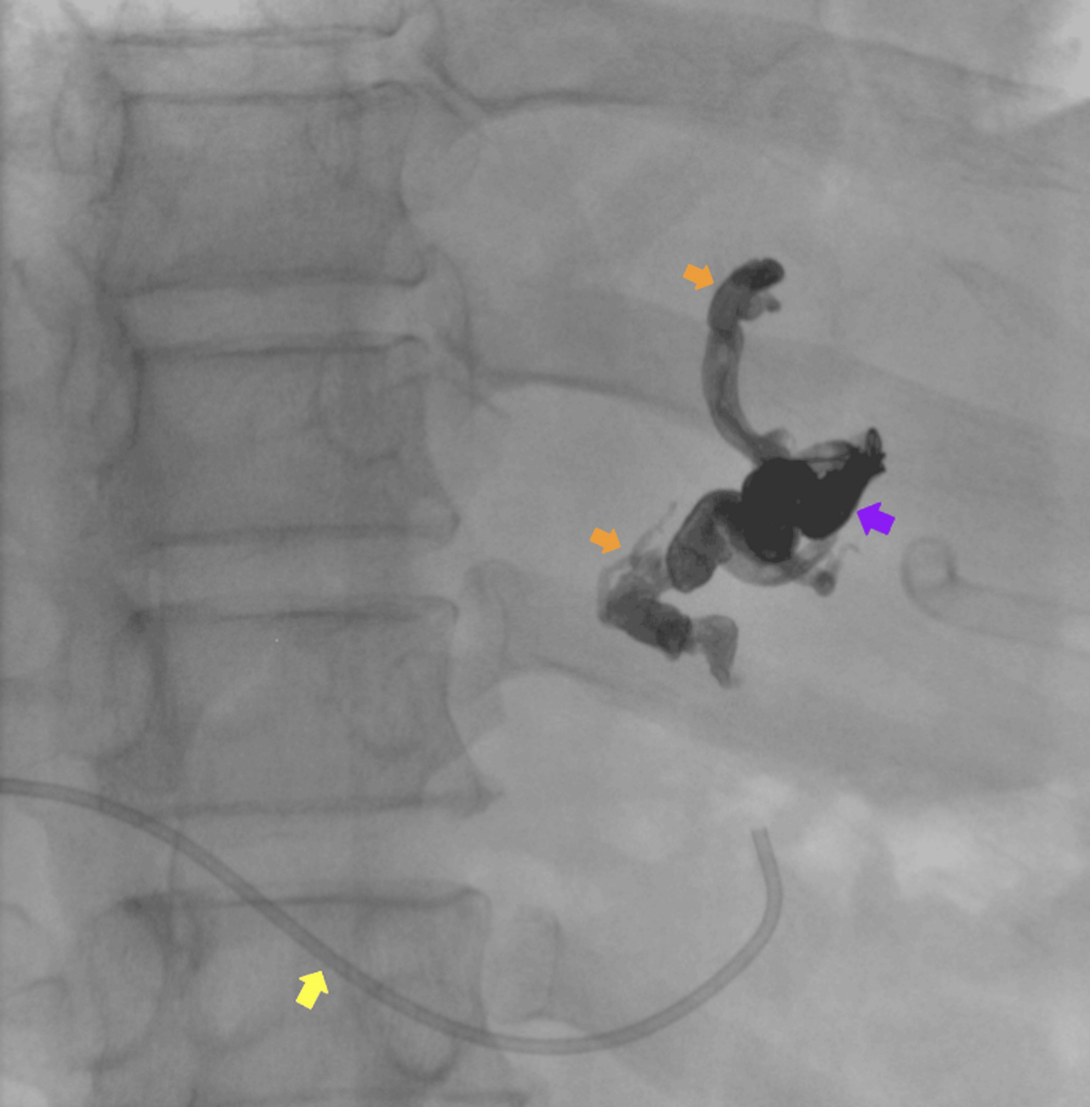
Gastrointestinal phlebography during Onyx and coil deployment Using a Progreat microcatheter, Onyx was deployed over the previously placed coils to create a seal. Yellow arrow: transhepatic Cobra catheter. Orange arrows: Onyx (dark gray) over coils.

**Figure 6:**
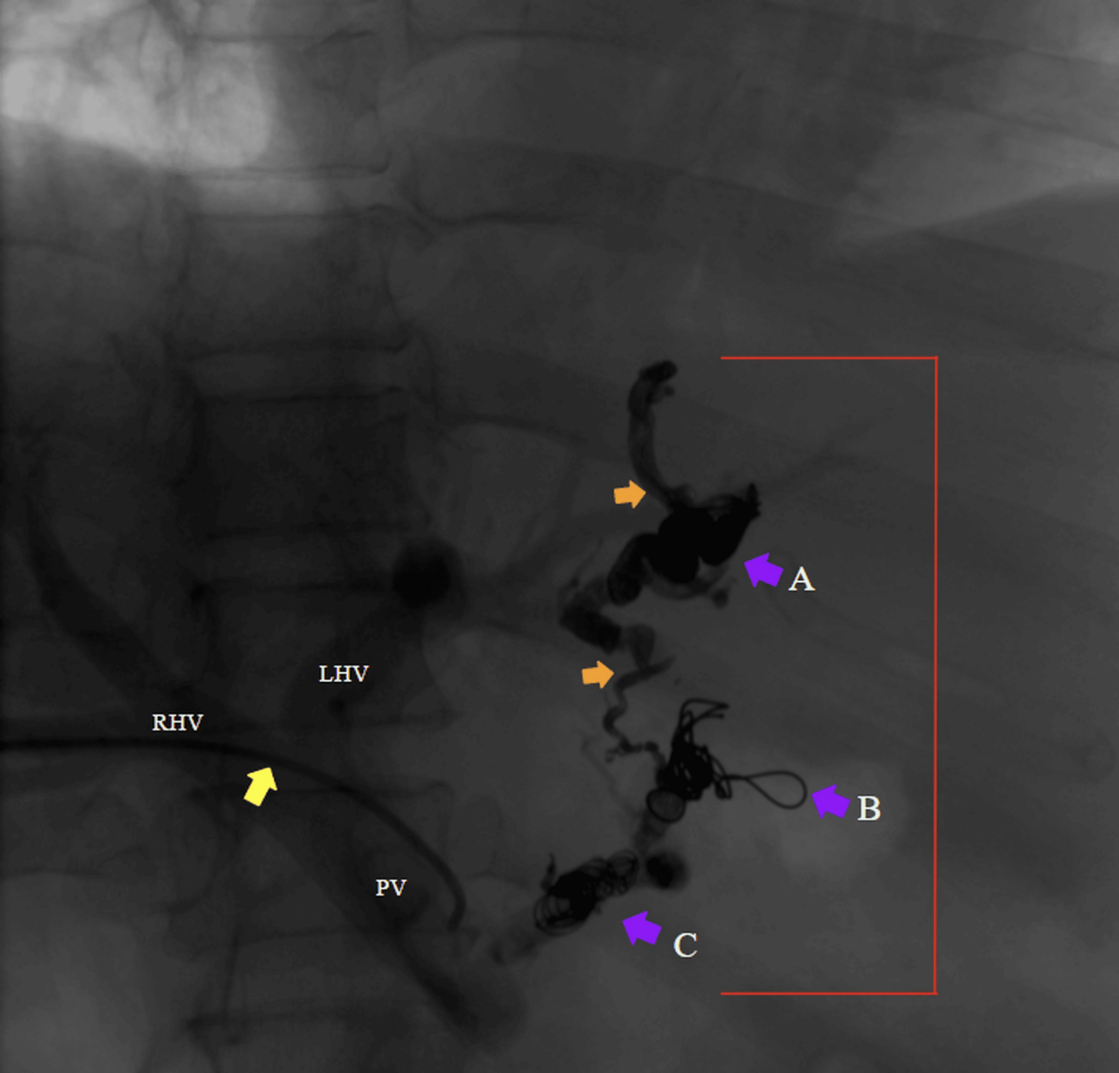
Gastrointestinal phlebography showing Onyx and three coils in position The image demonstrates three coils correctly positioned along the gastroesophageal variceal tract, with Onyx deposited over the coils. Purple arrows: (A) first coil deployed, (B) second coil deployed, and (C) third coil deployed, arranged from distal to proximal. Orange arrows: Onyx (dark gray) over each coil. Yellow arrow: transhepatic Cobra catheter. Red lines: gastroesophageal varices. LHV: left hepatic vein. RHV: right hepatic vein. PV: portal vein.

**Figure 7:**
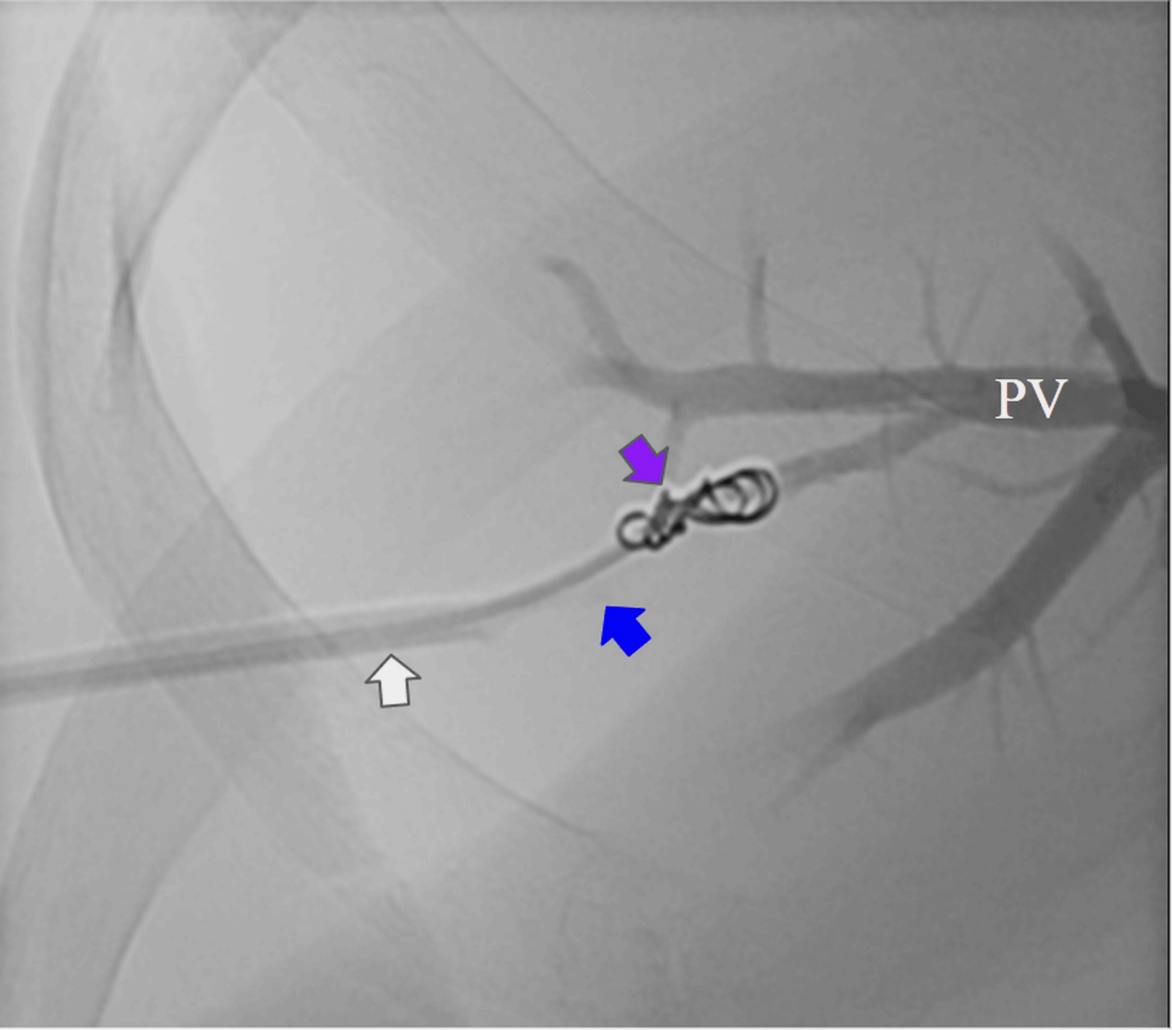
Transhepatic portography showing coil embolization of the puncture tract Coil embolization of the proximal transhepatic puncture tract was performed to achieve hemostasis and prevent access-site bleeding. PV: portal veins. Purple arrow: last coil deployed. Blue arrows: progreat microcatheter. White arrow: vascular introductor.

Three dominant variceal pedicles were selectively catheterized using 2.7-F Progreat™ microcatheters (Terumo, Tokyo, Japan) (Figures 3-4). Embolization was performed sequentially, advancing the microcatheter distally to create a dense coil cast, prevent Onyx migration into the pulmonary circulation, and reduce the risk of pulmonary embolism (Figure 4). The first pedicle was occluded with detachable coils (8 × 30 mm, 13 × 30 mm, and two 8 × 20 mm coils) (TJW Medical, China), followed by Onyx installation (Medtronic, Minneapolis, MN, USA) (Figure 5). A second variceal channel was embolized using two coils (6 × 20 mm and 10 × 20 mm). The final pedicle was occluded with a (5 × 8 mm) coil and supplemental Onyx (Figures 5-6). To conclude the procedure, coil embolization forming a cast was performed at the most proximal portion of the puncture tract to promote hemostasis and prevent access-site bleeding (Figure 7).

**Figure 8:**
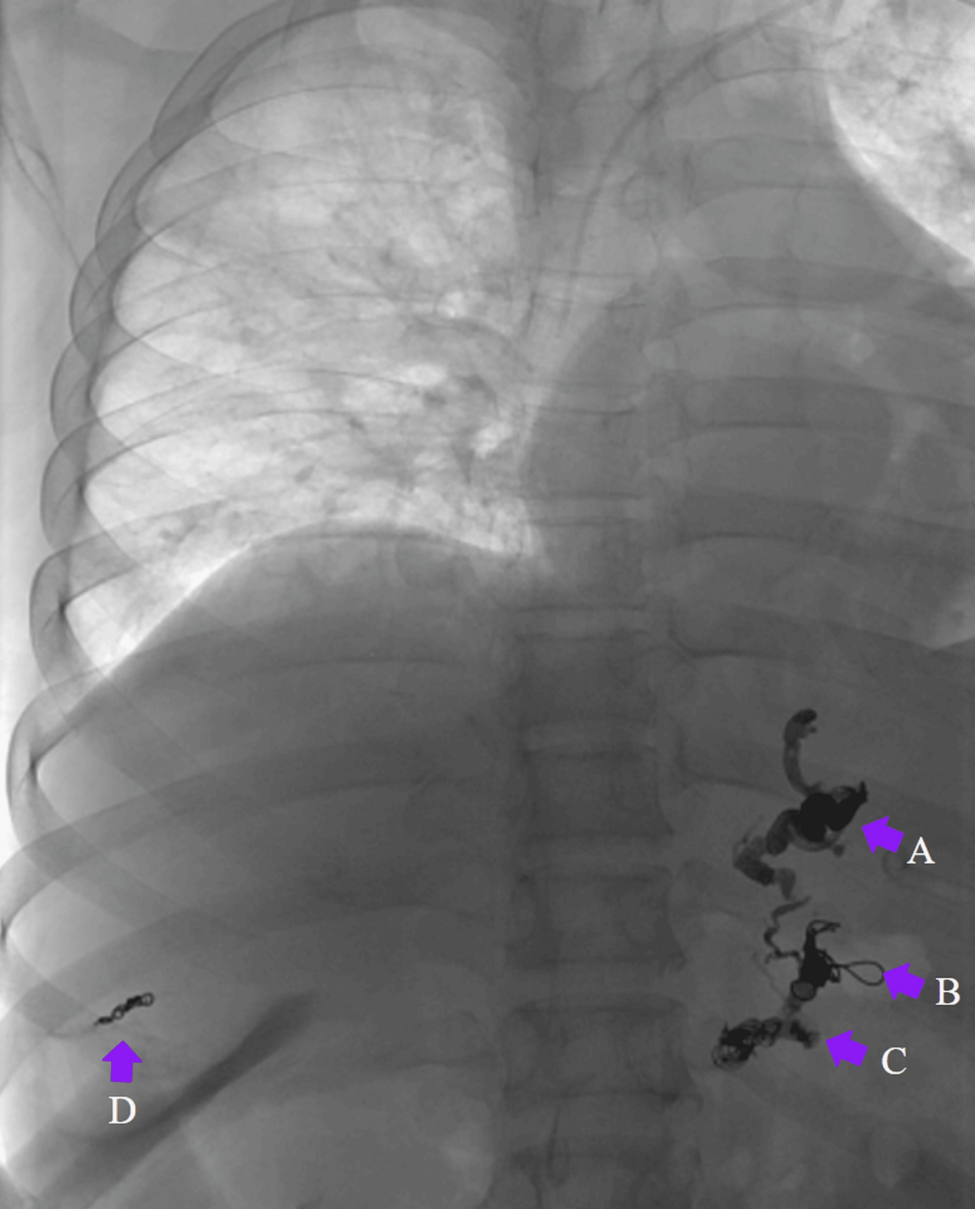
Final control arteriography demonstrating adequate embolization and successful bleeding control of gastroesophageal varices Final control arteriography demonstrating adequate embolization and successful control of gastroesophageal variceal bleeding. Purple arrows: coil deployment sequence: (A) first coil, (B) second coil, (C) third coil, and (D) final coil.

Completion portography confirmed complete occlusion of variceal inflow with no non-target embolization (Figure 8). The sheaths were removed after the transhepatic access tract was embolized using coil-assisted occlusion and a Gelfoam-Axiostat® patch (Advamedica, MA, USA) to achieve hemostasis. No intraprocedural complications occurred.

## Discussion

Cryptogenic cirrhosis accounts for approximately 5-10% of all cirrhosis cases [6]. When the portal pressure gradient exceeds 10 mmHg, esophageal or gastric varices develop, and the risk of rupture increases with rising portal pressure [6,7].

In the present case, the patient had decompensated cryptogenic cirrhosis with ascites and bleeding gastroesophageal varices type 1 [8]. Risk stratification showed Child-Pugh class B (eight points; total bilirubin 0.74 mg/dL, albumin 2.85 g/dL, international normalized ratio (INR) 1.55) and a MELD-Na score of 11 (no dialysis; creatinine 0.39 mg/dL, total bilirubin 0.74 mg/dL, INR 1.55, sodium 137 mmol/L), corresponding to an estimated one-year survival of 81% and a three-month mortality of approximately 6% [9].

According to guidance from the American Association for the Study of Liver Diseases (AASLD) and the American College of Radiology, patients with Child-Pugh B and active bleeding should receive vasoactive drugs, antibiotic prophylaxis, and urgent endoscopy within the first 12 hours [8,10,11]. These guidelines also recommend considering early TIPS depending on the initial response to endoscopic variceal ligation (EVL) [7,8,10]. The patient received terlipressin, a long-acting V1 receptor agonist that reduces splanchnic blood flow and portal pressure, improving early hemostasis for endoscopic management [8]. However, in many low- and middle-income settings, continuous access to therapeutic endoscopy is not available, limiting adherence to recommended treatment timelines (<12 hours) [3]. Although EVL is the standard endoscopic approach for acute esophageal variceal bleeding, it has limitations, including restricted availability, the need for repeated sessions, and risk of early bleeding [12]. Accordingly, recent guidelines recognize endovascular therapy as an alternative when endoscopy is unavailable or unsuccessful [8].

In this context, PTVE was performed via direct portal venous access. This technique is supported by emerging evidence demonstrating utility when endoscopy is not feasible or as a bridge to TIPS or portal decompression [13,14]. The combination of coils and Onyx (ethylene-vinyl alcohol copolymer) provided a more durable and stable occlusion. The addition of a non-adhesive liquid embolic may improve hemostatic stability, reduce variceal reperfusion, and lower the risk of rebleeding compared with coils alone [5,15]. Additionally, Onyx allows a complete embolization of small tributary veins [16].

A recent study evaluating PTVE suggests that the intervention may be safe and effective, with a low complication rate, and may be considered in situations where standard strategies are challenging to implement [5]. Consistent with that study, we used a non-balloon-assisted technique using Cobra catheters and Progreat microcatheters to avoid the challenges and technical failures associated with balloon catheter manipulation through tortuous variceal anatomy [5]. We also used coils combined with a non-adhesive liquid embolic (Onyx), rather than sclerosing agents, which have been associated with hemolysis and renal impairment, and in contrast to the comparative study that used coils with cyanoacrylate [5]. Unlike sclerosants, which may migrate due to their low viscosity and dependence on chemical thrombosis, Onyx precipitates gradually to form a solid cast, allowing controlled deployment with significantly reduced risk of non-target migration, particularly when used in combination with coils [5].

Although most of the experience with variceal embolization involves gastric or ectopic varices, systematic reviews support the feasibility and effectiveness of Onyx coils for gastrointestinal hemorrhage, including esophageal varices [15,16]. In other hemorrhage settings, Onyx has demonstrated safety and efficacy with low complication and rebleeding rates [16]. While direct evidence for esophageal varices is limited, extrapolation from available data from gastric varices suggests that this technique may be appropriate in selected cases at experienced centers, particularly when EVL is not feasible [13,17]. As reported in the literature, the combined hemodynamic effect of coils (flow reduction) and Onyx (complete variceal occlusion) shortened procedural time and minimized complications [5]. In our case, completion portography confirmed full occlusion of the variceal pedicles without adverse events.

Although PTVE is an established technique, this case is distinctive owing to the combination of factors: emergency presentation with hypovolemic shock, cryptogenic cirrhosis with gastroesophageal varices, lack of immediate endoscopic or TIPS availability, and a combined coil and Onyx embolization strategy via selective transhepatic access. Most published reports focus on gastric or ectopic varices or describe embolization as adjunctive therapy. In contrast, this case demonstrates the feasibility of percutaneous transhepatic embolization as a primary life-saving intervention for esophageal variceal bleeding in a resource-limited setting, highlighting both its technical complexity and its clinical relevance as bridge therapy.

This case is clinically relevant because evidence supporting PTVE for esophageal varices remains limited, with most studies focusing on gastric varices, particularly cardiofundal types [4]. Furthermore, few publications describe its use as a primary emergency intervention in patients with cryptogenic cirrhosis and hemodynamic instability in resource-limited settings [4]. Current AASLD guidelines support endovascular therapy as an alternative in bleeding varices, particularly in conjunction with TIPS or through technical variants such as balloon-occluded retrograde transvenous obliteration (BRTO) or balloon-occluded antegrade transvenous obliteration (BATO) for gastric varices [8]. These techniques (BRTO/BATO) may be considered in patients with hepatic encephalopathy, high MELD-Na score, or when TIPS is contraindicated [4].

Ideally, this patient met criteria for early TIPS, given Child-Pugh class B cirrhosis with active bleeding and ascites, and evidence supports TIPS placement within 72 hours in selected patients to reduce rebleeding and improve outcomes [4,8,13,18,19]. The goal of this procedure is to reduce portal pressure by at least 20% relative to baseline and has been shown to benefit survival and prevent recurrent rebleeding [4,8]. However, limited access to specialized endoscopy, gastroenterology, and interventional radiology during the acute phase delays definitive therapy.

Under these circumstances, PTVE provided rapid, safe, and effective hemostasis while serving as a bridge during patient stabilization and referral. Target hemoglobin goals (7-8 g/dL) were achieved during the initial management period [3,20].

This case underscores the critical role of interventional radiology in resource-limited settings, where delays in endoscopy or TIPS can significantly impact survival. It also contributes to the scarce literature on PTVE for esophageal varices, supporting its potential as an alternative when standard therapies are not feasible [5]. Finally, it highlights the importance of a multidisciplinary approach and the need to consider early endovascular strategies in the management of acute variceal bleeding when conventional options are unavailable.

## Conclusions

PTVE can provide rapid hemostatic control and hemodynamic stabilization in acute variceal bleeding when first-line therapies such as urgent endoscopy or TIPS are unavailable or delayed. This case supports PTVE as a feasible bridge strategy in time-sensitive or resource-limited settings. Although current evidence remains limited and is primarily based on small series and case reports, this approach may be considered in selected patients at centers with appropriate expertise and multidisciplinary support.

## References

[1] Ishikawa T, Sasaki R, Nishimura T (2019). A novel therapeutic strategy for esophageal varices using endoscopic treatment combined with splenic artery embolization according to the Child-Pugh classification. PLoS One.

[2] Zuckerman MJ, Elhanafi S, Mendoza Ladd A (2022). Endoscopic treatment of esophageal varices. Clin Liver Dis.

[3] Hwang JH, Shergill AK, Acosta RD (2014). The role of endoscopy in the management of variceal hemorrhage. Gastrointest Endosc.

[4] Davis JP, Lim JK, Francis FF, Ahn J (2025). Aga clinical practice update on management of portal vein thrombosis in patients with cirrhosis: expert review. Gastroenterology.

[5] Tie J, Yuan X, Zhu Y (2024). Efficacy and safety of variceal embolization for primary prophylaxis in cirrhosis patients with challenges in standard treatments: preliminary results. Front Med (Lausanne).

[6] Nalbantoglu I, Jain D (2019). Cryptogenic cirrhosis: old and new perspectives in the era of molecular and genomic medicine. Semin Diagn Pathol.

[7] de Franchis R, Bosch J, Garcia-Tsao G, Reiberger T, Ripoll C (2022). Baveno VII - renewing consensus in portal hypertension. J Hepatol.

[8] Kaplan DE, Ripoll C, Thiele M, Fortune BE, Simonetto DA, Garcia-Tsao G, Bosch J (2024). AASLD practice guidance on risk stratification and management of portal hypertension and varices in cirrhosis. Hepatology.

[9] Moore O, Ma WS, Read S, George J, Ahlenstiel G (2025). The unwell patient with advanced chronic liver disease: when to use each score?. BMC Med.

[10] Pinchot JW, Kalva SP, Majdalany BS (2021). ACR appropriateness criteria® radiologic management of portal hypertension. J Am Coll Radiol.

[11] Simonetto DA, Liu M, Kamath PS (2019). Portal hypertension and related complications: diagnosis and management. Mayo Clin Proc.

[12] Gralnek IM, Camus Duboc M, Garcia-Pagan JC (2022). Endoscopic diagnosis and management of esophagogastric variceal hemorrhage: European Society of Gastrointestinal Endoscopy (ESGE) guideline. Endoscopy.

[13] Dervishi M, Stevens A, Sutter C, Chiong I, Davidson J (2025). Endovascular management of bleeding ectopic varices. Tech Vasc Interv Radiol.

[14] Patel RK, Tripathy T, Mukund A, Panigrahi MK, Pattnaik B, Behera S, Nayak HK (19). Interventional Management of variceal bleeding: techniques and emerging concepts. Dig Dis Interv.

[15] Kolber MK, Shukla PA, Kumar A, Silberzweig JE (2015). Ethylene vinyl alcohol copolymer (onyx) embolization for acute hemorrhage: a systematic review of peripheral applications. J Vasc Interv Radiol.

[16] Wang L, Zhou JL, Yang N, Zhang GN, Lu JY, Xiao Y, Qiu HZ (2015). Ectopic variceal bleeding from colonic stoma: two case reports. Medicine (Baltimore).

[17] Lee IJ (2018). Outcomes and complications of embolization for gastrointestinal bleeding. Gastrointest Interv.

[18] Bettinger D, Thimme R, Schultheiß M (2022). Implantation of transjugular intrahepatic portosystemic shunt (TIPS): indication and patient selection. Curr Opin Gastroenterol.

[19] Cannella R, Tselikas L, Douane F, Cauchy F, Rautou PE, Duran R, Ronot M (2022). Imaging-guided interventions modulating portal venous flow: evidence and controversies. JHEP Rep.

[20] Villanueva C, Colomo A, Bosch A (2013). Transfusion strategies for acute upper gastrointestinal bleeding. N Engl J Med.

